# The Ketogenic Diet and Hyperbaric Oxygen Therapy Prolong Survival in Mice with Systemic Metastatic Cancer

**DOI:** 10.1371/journal.pone.0065522

**Published:** 2013-06-05

**Authors:** Angela M. Poff, Csilla Ari, Thomas N. Seyfried, Dominic P. D’Agostino

**Affiliations:** 1 Department of Molecular Pharmacology and Physiology, University of South Florida, Tampa, Florida, United States of America; 2 Department of Biology, Boston College, Chestnut Hill, Massachusetts, United States of America; China Medical University, Taiwan

## Abstract

**Introduction:**

Abnormal cancer metabolism creates a glycolytic-dependency which can be exploited by lowering glucose availability to the tumor. The ketogenic diet (KD) is a low carbohydrate, high fat diet which decreases blood glucose and elevates blood ketones and has been shown to slow cancer progression in animals and humans. Abnormal tumor vasculature creates hypoxic pockets which promote cancer progression and further increase the glycolytic-dependency of cancers. Hyperbaric oxygen therapy (HBO_2_T) saturates tumors with oxygen, reversing the cancer promoting effects of tumor hypoxia. Since these non-toxic therapies exploit overlapping metabolic deficiencies of cancer, we tested their combined effects on cancer progression in a natural model of metastatic disease.

**Methods:**

We used the firefly luciferase-tagged VM-M3 mouse model of metastatic cancer to compare tumor progression and survival in mice fed standard or KD *ad libitum* with or without HBO_2_T (2.5 ATM absolute, 90 min, 3x/week). Tumor growth was monitored by *in vivo* bioluminescent imaging.

**Results:**

KD alone significantly decreased blood glucose, slowed tumor growth, and increased mean survival time by 56.7% in mice with systemic metastatic cancer. While HBO_2_T alone did not influence cancer progression, combining the KD with HBO_2_T elicited a significant decrease in blood glucose, tumor growth rate, and 77.9% increase in mean survival time compared to controls.

**Conclusions:**

KD and HBO_2_T produce significant anti-cancer effects when combined in a natural model of systemic metastatic cancer. Our evidence suggests that these therapies should be further investigated as potential non-toxic treatments or adjuvant therapies to standard care for patients with systemic metastatic disease.

## Introduction

Metastasis is a complex phenomenon in which cancer cells spread from a primary tumor to establish foci in a distal tissue and is responsible for 90 percent of cancer-related deaths [1]. The specific changes which mediate metastasis remain unclear; however, the process generally involves local tumor growth, invasion through the basement membrane and surrounding tissue, intravasation into the lymphatics or blood vessels, dissemination and survival in circulation, extravasation from the vasculature, and re-establishment of tumors at distal tissues. While many primary tumors can be controlled with conventional therapies like surgery, chemotherapy, and radiation, these treatments are often ineffective against metastatic disease and in some cases may promote cancer progression and metastasis [2], [3], [4]. There is a substantial need for novel therapies effective against metastatic cancer.

Perhaps the most important limiting factor in the development of new treatments for metastatic cancer is the lack of animal models that accurately reflect the true nature of metastatic disease. Xenograft models of human cancers in immunodeficient mice are inadequate as the immune system is highly involved in cancer development and progression. Indeed, most tumor models grown as xenografts in immune compromised mice fail to metastasize [5], [6]. Tail vein injection models of metastatic cancer eliminate the important steps of local tissue invasion and intravasation into the vasculature, again failing to represent the true disease phenotype. The VM-M3 model of metastatic cancer is a novel murine model that closely mimics the natural progression of invasion and metastasis [7], [8]. The VM-M3 tumor arose spontaneously in the brain of a mouse of the VM/Dk inbred strain and expresses multiple growth characteristics of human glioblastoma multiforme with macrophage/microglial properties [7], [9]. When implanted subcutaneously, VM-M3 cells rapidly metastasize to all major organ systems, notably the liver, lung, kidney, spleen, brain, and bone marrow. Systemic metastasis has also been repeatedly documented in human glioblastoma multiforme (GBM), which has been linked to the macrophage/microglial characteristics of the tumor [9]. The tumor was adapted to cell culture and transfected with the firefly luciferase gene to allow for easy monitoring of tumor growth *in vivo* [10]. The VM-M3 model of metastatic cancer has a distinct advantage over other metastatic models because it spreads naturally in an immunocompetent host, mimicking the natural cancer microenvironment. Cisplatin and methotrexate, two commonly-used chemotherapy agents, inhibit metastatic spread in the VM-M3 model of metastatic cancer similarly to their effects in humans, further supporting the model’s representation of the true disease state [8], . For these reasons, the VM-M3 model of metastatic cancer was used for this study.

Abnormal energy metabolism is a consistent feature of most tumor cells across all tissue types [14]. In the 1930 s, Otto Warburg observed that all cancers expressed high rates of fermentation in the presence of oxygen [15]. This feature, known as The Warburg Effect, is linked to mitochondrial dysfunction and genetic mutations within the cancer cell [14], [16], [17]. These defects cause cancers to rely heavily on glucose for energy, a quality that underlies the use of fluorodeoxyglucose-PET scans as an important diagnostic tool for oncologists [18]. Ketogenic diets are high fat, low carbohydrate diets that have been used for decades to treat patients with refractory epilepsy [19]. Ketogenic diets also suppress appetite naturally thus producing some body weight loss [19], [20], [21], [22]. Dietary energy reduction (DER) lowers blood glucose levels, limiting the energy supply to cancer cells, while elevating circulating blood ketone levels [6]. Ketone bodies can serve as an alternative energy source for those cells with normal mitochondrial function [23], [24], but not for cancer cells [25]. DER has been shown to have anti-tumor effects in a variety of cancers, including brain, prostate, mammary, pancreas, lung, gastric, and colon [14], [26], [27], [28], [29], [30], [31], [32], [33], [34]. DER produces anti-cancer effects through several metabolic pathways, including inhibition of the IGF-1/PI3K/Akt/HIF-1α pathway which is used by cancer cells to promote proliferation and angiogenesis and inhibit apoptosis [35], [36], [37], [38], [39], [40], [41], [42]. Additionally, DER induces apoptosis in astrocytoma cells, while protecting normal brain cells from death through activation of adenosine monophosphate kinase (AMPK) [43].

Tumors possess abnormal vasculature which blocks adequate tissue perfusion, leading to the presence of hypoxic regions that promote chemotherapy and radiation resistance [44], [45], [46], [47]. In fact, hypoxic cancer cells are three-times more resistant to radiation therapy than are well-oxygenated cells [48]. In addition to decreasing the efficacy of standard care, tumor hypoxia activates a number of oncogene pathways, largely through the HIF-1 transcription factor, which promote tumor growth, metastasis, angiogenesis, and inhibit apoptosis [49], [50].

Hyperbaric oxygen therapy (HBO_2_T) involves administration of 100% oxygen at elevated pressure (greater than sea level, or 1 ATA). HBO_2_T increases plasma oxygen saturation which facilitates oxygen delivery to the tissue independent of hemoglobin O_2_ saturation [51]. The potential benefit of using HBO_2_T to combat the cancer-promoting effects of tumor hypoxia is clear. HBO_2_T alone has been shown to inhibit tumor growth, reduce tumor blood vessel density, and induce the preferential expression of anti-cancer genes in rat models of mammary tumors [52]. Additionally, radiation and many chemotherapy drugs work by producing free radicals within the tumors, leading to cell death. HBO_2_T enhances tumor-cell production of reactive oxygen species which contributes to the synergistic effects of HBO_2_T as an adjuvant treatment to standard care. Indeed, HBO_2_T enhances the efficacy of both radiation and chemotherapy in animal models [53], [54], [55], [56], [57].

In normal tissues, decreased oxygen availability inhibits mitochondrial production of ATP, stimulating an up-regulation of glycolytic enzymes to meet energy needs by substrate level phosphorylation production of ATP. Thus, the cellular response to tumor hypoxia is mediated by several of the same pathways that are overly active in cancer cells with mitochondrial damage and high rates of aerobic glycolysis. This suggests that the ketogenic diet and HBO_2_T could target several overlapping pathways and tumorigenic behaviors of cancer cells. We hypothesized that these treatments would work synergistically to inhibit tumor progression. We suggest that the addition of these non-toxic adjuvant therapies to the current standard of care may improve progression free survival in patients with advanced metastatic disease.

## Materials and Methods

### Mice

Three breeding pairs of the VM/Dk strain of mice were used to establish and propagate a VM/Dk mouse colony in the University of South Florida (USF) Morsani College of Medicine Vivarium according to standard husbandry protocol. Forty adult male mice (10–18 weeks of age) were used for this study. All animal procedures were performed within strict adherence to the NIH Guide for the Care and Use of Laboratory and Animals and were approved by the USF Institutional Animal Care and Use Committee (IACUC; Protocol Number R4137).

### Cell Culture

VM-M3/Fluc cells were received as a gift from T.N. Seyfried, Boston College, where they were created from a spontaneous tumor in a VM/Dk mouse and adapted to cell culture [7]. VM-M3/Fluc cells were transduced with a lentivirus vector containing the firefly luciferase gene under control of the cytomegalovirus promoter (VM-M3/Fluc) as previously described [7]. The VM-M3/Fluc cells were cultured in Eagle’s Minimum Essential Medium with 2 mM L-glutamine (ATCC, Manassas, VA), 10% fetal bovine serum (Invitrogen, Grand Island, NY), 1% penicillin-streptomycin (Gibco, Invitrogen) and high glucose (25 mM D-glucose, Fisher Scientific, Waltham, MA). Cells were cultured in a CO_2_ incubator at 37°C in 95% air and 5% CO_2_.

### Subcutaneous Tumor Implantation

On day 0, VM-M3/Fluc cells (1 million cells in 300 µL PBS) were implanted, s.c., into the abdomen of VM/Dk mice using a 27 gage needle. Inoculation results in rapid and systemic metastasis to most major organs, namely liver, kidneys, spleen, lungs, and brain as previously described [7].

### Diet Therapy

On the day of tumor inoculation, mice were randomly assigned to one of four groups: SD (Control); SD+HBO_2_T; KD; or KD+HBO_2_T. Mice in the SD group were fed standard rodent chow (2018 Teklad Global 18% Protein Rodent Diet, Harlan) *ad libitum.* Mice in the KD group received KD-Solace ketogenic diet *ad libitum*. KD-Solace is a commercially available ketogenic diet powder (KetoGen, Solace Nutrition) and was mixed 1∶1 with H_2_O to form a solid paste. Macronutrient information for SD and KD-Solace are shown in Table 1. Diets were continuously replaced every other day to maintain freshness and allow mice to feed *ad libitum.*

**Table 1:** Macronutrient information of diets.

Macronutrient Information	Standard Diet	KD-Solace
% Cal from Fat	18.0	89.2
% Cal from Protein	24.0	8.7
% Cal from Carbohydrate	58.0	2.1
Caloric Density	3.1 Kcal/g	7.12 Kcal/g

### Hyperbaric Oxygen Therapy (HBO_2_T)

Mice undergoing HBO_2_T received 100% O_2_ for 90 minutes at 1.5 ATM gauge (2.5 ATM absolute) three times per week (M, W, F) in a hyperbaric chamber (Model 1300B, Sechrist Industries, Anaheim, CA).

### Glucose, Ketone, and Weight Measurements

Every 7 days, blood was collected from the tail using approved methods. Glucose was measured using the Nova Max® Plus™ Glucose and β-Ketone Monitoring System (Nova Biomedical, Waltham, MA), and β-hydroxybutyrate was measured using the Precision Xtra™ Blood Glucose & Ketone Monitoring System (Abbott Laboratories, Abbott Park, IL).

Mice were weighed between 1 and 3 pm twice a week for the duration of the study using the AWS-1KG Portable Digital Scale (AWS, Charleston, SC).

### Bioluminescent Imaging and Tumor Growth Analysis

Tumor growth was monitored as a measure of bioluminescent signaling using the Xenogen IVIS Lumina system (Caliper LS, Hopkinton, MA). Data acquisition and analysis was performed using the Living Image® software (Caliper LS). Approximately 15 minutes prior to *in vivo* imaging, the mice received an i.p. injection of D-Luciferin (50 mg/kg) (Caliper LS). Bioluminescent signal was obtained using the IVIS Lumina cooled CCD camera system with a 1 sec exposure time. As only the cancer cells contained the luciferase gene, bioluminescent signal (photons/sec) of the whole animal was measured and tracked over time as an indicator of metastatic tumor size and spread.

### Survival Analysis

Throughout the study, health and behavior of the mice were assessed daily. Mice were humanely euthanized by CO_2_ asphyxiation according to IACUC guidelines upon presentation of defined criteria (tumor-associated ascites, diminished response to stimuli, lethargy, and failure to thrive), and survival time was recorded.

### Statistics

Survival data was analyzed by the Kaplan-Meier and Logrank Tests for survival distribution. Mean survival times were analyzed by two-tailed student’s t-tests. Bioluminescent signal as a measure of tumor size was analyzed by two-tailed student’s t-tests. Blood measurements were analyzed by One Way ANOVA with Kruskal Wallis Test and Dunn’s Multiple Comparison Test post hoc. Differences in percent weight change were analyzed by One Way ANOVA with Tukey’s Multiple Comparison Test post hoc. Correlation between blood glucose, body weight change, and survival times were analyzed by linear regression analysis. Results were considered significant when p<0.05.

## Results

### Combining the KD with HBO_2_T Prolonged Survival in Mice with Metastatic Cancer

**Figure 1:**
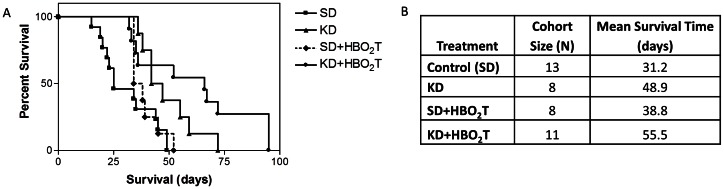
The KD and HBO_2_T increases survival time in mice with systemic metastatic cancer. (**A**) Kaplan-Meier survival plot of study groups. Animals receiving KD and KD+HBO_2_T showed significantly longer survival compared to control animals (p = 0.0194 and p = 0.0035, respectively; Kaplan-Meier and LogRank Tests for survival distribution). (**B**) Treatment group cohort size and mean survival times shown. KD mice exhibited a 56.7% increase in mean survival time compared to controls (p = 0.0044; two-tailed student’s t-test); KD+HBO_2_T mice exhibited a 77.9% increase in mean survival time compared to controls (p = 0.0050; two-tailed student’s t-test). Results were considered significant when p<0.05.

KD and KD+HBO_2_T treated mice demonstrated a statistically different survival curve by Logrank Test with an increase in survival time compared to control animals (p = 0.0194 and p = 0.0035, respectively; Figure 1A). KD fed and KD+HBO_2_T animals also showed a significant increase in mean survival time compared to control animals by the two-tailed student’s t-test (p = 0.0044 and p = 0.0050, respectively; Figure 1B). Although previous studies have reported that HBO_2_T alone can increase survival time in animals with various cancers [52], [54], [58], [59], we did not see an effect on survival in mice receiving SD+HBO_2_T. Control (SD) mice had a mean survival time of 31.2 days whereas SD+HBO_2_T mice had a non-statistically different mean survival of 38.8 days (Figure 1B). The KD alone increased mean survival time by approximately 17 days (56.7%), and when combined with HBO_2_T, mice exhibited an increase in mean survival time of approximately 24 days (77.9%) (Figure 1B). This finding strongly supports the efficacy of the KD and HBO_2_T as therapies to inhibit tumor progression and prolong survival in animals with metastatic cancer.

### The KD and HBO_2_T Decreased Tumor Bioluminescence

**Figure 2:**
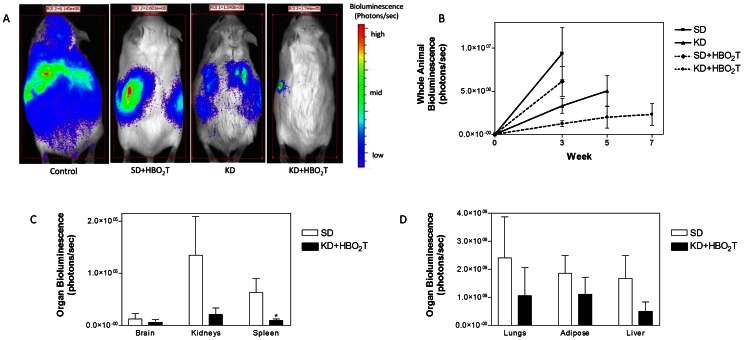
Tumor bioluminescence in mice. Tumor growth was slower in mice fed the KD than in mice fed the SD. (A) Representative animals from each treatment group demonstrating tumor bioluminescence at day 21 after tumor cell inoculation. Treated animals showed less bioluminescence than controls with KD+HBO2T mice exhibiting a profound decrease in tumor bioluminescence compared to all groups. (B) Total body bioluminescence was measured weekly as a measure of tumor size; error bars represent ±SEM. KD+HBO2T mice exhibited significantly less tumor bioluminescence than control animals at week 3 (p = 0.0062; two-tailed student‚s t-test) and an overall trend of notably slower tumor growth than controls and other treated animals throughout the study. (C,D) Day 21 ex vivo organ bioluminescence of SD and KD+HBO2T animals (N = 8) demonstrated a trend of reduced metastatic tumor burden in animals receiving the combined therapy. Spleen bioluminescence was significantly decreased in KD+HBO2T mice (*p = 0.0266; two-tailed student‚s t-test). Results were considered significant when p<0.05.

Bioluminescent signal was tracked as a measure of tumor size throughout the study. Animals receiving the KD alone or in combination with HBO_2_T demonstrated a notable trend of slower tumor growth over time. This trend was more pronounced in KD+HBO_2_T mice and reflected the increase in survival time seen in these animals (Figures 1, 2). The difference in mean tumor size between KD+HBO_2_T and control animals at week 3 was statistically significant (p = 0.0062; Figure 2B). Day 21 e*x vivo* organ bioluminescence of KD+HBO_2_T mice demonstrated a trend of reduced metastatic tumors in animals compared to the SD group (Figure 2). Spleen bioluminescence was significantly decreased in KD+HBO_2_T mice (p = 0.0266).

### The KD Lowered Blood Glucose, Elevated Blood Ketones, and Decreased Body Weight

**Figure 3:**
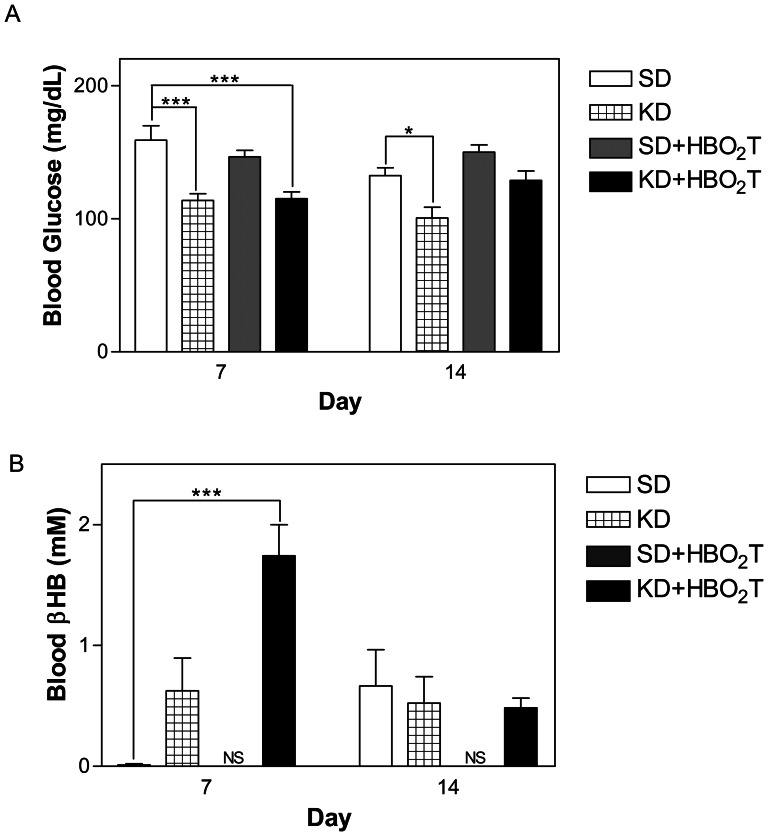
Blood glucose and β-hydroxybutyrate levels in animals. (A) KD-fed mice showed lower blood glucose than controls on day 7 (***p<0.001). Animals in the KD study group had significantly lower blood glucose levels than controls on day 14 (*p<0.05). (B) KD+HBO2T mice had significantly higher blood ketones than controls on day 7 (***p<0.001). Error bars represent ±SEM. Blood analysis was performed with One Way ANOVA with Kruksal Wallis Test and Dunn‚s Multiple Comparison Test post hoc; results were considered significant when p<0.05.

**Figure 4:**
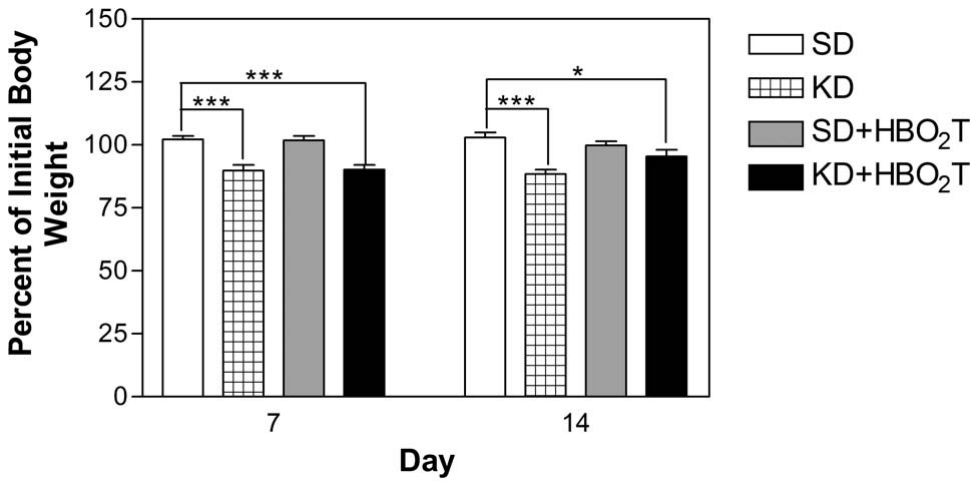
Animal weight. Body weight was measured twice a week. Graph indicates average percent of initial body weight animals at days 7 and 14. KD and KD+HBO2T mice lost approximately 10% of their body weight by day 7 and exhibited a significant difference in percent body weight change compared to control animals (*p<0.05; ***p<0.001). Error bars represent ±SEM.

**Figure 5:**
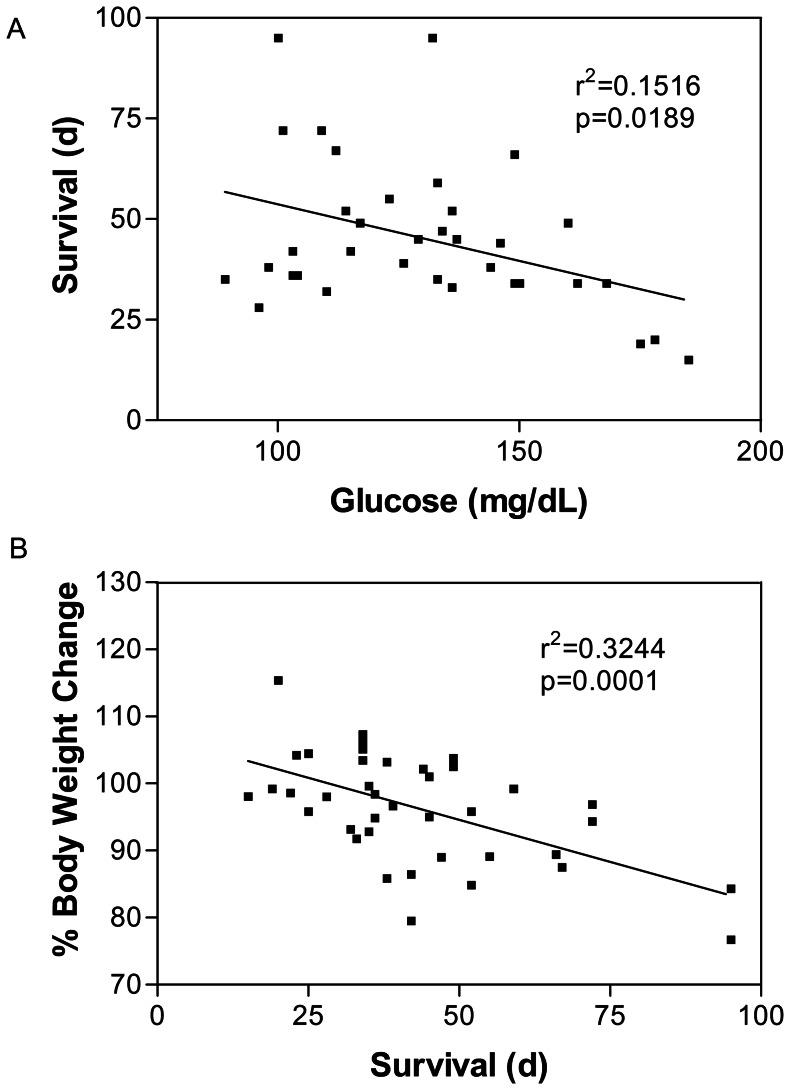
Glucose and weight change are correlated to survival. Linear regression analysis revealed a significant correlation between day 7 blood glucose and percent body weight change with survival (p = 0.0189 and p = 0.0001, respectively). Results were considered significant when p<0.05.

Prior to the study, initial blood glucose, ketone, and body weights were similar among the groups (data not shown). Blood glucose levels were lower in the KD-treated mice than in the SD-treated mice by day 7 (p<0.001; Figure 3). While all KD-fed mice demonstrated a trend of elevated blood ketone levels throughout the duration of the study, only the KD+HBO_2_T animals showed significantly increased ketones compared to controls on day 7 (p<0.001; Figure 3). By day 7, KD-fed mice lost approximately 10% of their initial body weight and maintained that weight for the duration of the study (Figure 4). Day 7 blood glucose and percent body weight change were significantly correlated to survival time (p = 0.0189 and p = 0.0001, respectively; Figure 5).

## Discussion

Nearly a century after Otto Warburg reported the abnormal energy metabolism of cancer cells, renewed interest in the field has elucidated a plethora of novel therapeutic targets. Two promising treatments involve the use of HBO_2_T to reverse the cancer-promoting effects of tumor hypoxia and the use of the KD to limit the availability of glycolytic substrates to glucose-addicted cancer cells. Both therapies have been previously reported to possess anti-cancer effects [14], [54], [58], [60]. Since these treatments are believed to work by targeting several overlapping mechanisms, we hypothesized that combining these non-toxic treatments would provide a powerful, synergistic anti-cancer effect. Furthermore, since metastasis is responsible for the overwhelming majority of cancer-related deaths, we tested the efficacy of these conjoined therapies on the VM-M3 mouse model of metastatic cancer [7], [10].

We found that the KD fed *ad libitum* significantly increased mean survival time in mice with metastatic cancer (p = 0.0194; Figure 1). It is important to note that KD-fed animals lost approximately 10% of their body weight over the course of the study (Figure 4). It is well established that low carbohydrate, high fat ketogenic diets can cause body weight loss in overweight humans [21], [22], [61]. Ketogenic diets are also known to have an appetite suppressing effect which may contribute to body weight loss [20]. Along with appetite suppression, a potential contributing factor to the observed body weight loss is the possibility that mice found the KD to be less palatable and were self-restricting caloric intake. As calorie restriction is known to elicit profound anti-cancer effects, the ketogenic diet may inhibit cancer progression in part by indirect dietary energy restriction [6], [38]. Fine and colleagues recently used a very low carbohydrate KD to promote stable disease or partial remission in patients with advanced metastatic cancer [62]. Fine’s study demonstrated a correlation between blood ketones and response to the diet therapy, suggesting that ketone elevation itself also contributes to the anti-cancer efficacy of the KD.

As hypothesized, profound anti-cancer effects were observed in our metastatic mouse model after combining the KD with HBO_2_T. Combining these therapies nearly doubled survival time in mice with metastatic cancer, increasing mean survival time by 24 days compared to control animals (p = 0.0050; Figure 1). The KD+HBO_2_T-treated mice exhibited significantly decreased bioluminescence compared to controls at week 3 (p = 0.0062) and a trend of decreased tumor growth rate throughout the study (Figure 2). By day 7, all animals on a ketogenic diet had significantly lower blood glucose levels than controls (Figure 3). As it has been shown that tumor growth is directly correlated to blood glucose levels [63], this decrease in blood glucose concentration likely contributed to the trend of decreased tumor bioluminescence and rate of tumor growth seen in KD-fed animals (Figure 2). Nebeling et al. demonstrated that the KD significantly decreased glucose uptake in pediatric brain tumor patients by FDG-PET analysis [64]. This clinical data suggests decreased glucose delivery to the tumor is a causal mechanism in KD treatment. All KD-fed mice showed a trend of elevated blood ketones throughout the study; however, only KD+HBO_2_T mice had significantly higher ketones than controls on day 7 (Figure 3). As ketones are metabolized exclusively within the mitochondria, cancer cells with damaged mitochondria are unable to adequately use them for energy. Many cancers do not express the Succinyl-CoA: 3-ketoacid CoA-Transferase (SCOT) enzyme which is required for ketone body metabolism [65], [66]. In fact, βHB administration prevents healthy hippocampal neurons but not glioma cells from glucose withdrawal-induced cell death [24]. Furthermore, ketone bodies have anti-cancer effects themselves, possibly through inhibition of glycolytic enzymes [67]. Skinner and colleagues demonstrated that acetoacetate and βHB administration inhibits brain cancer cell viability *in vitro* [25]. Thus, the elevated ketone levels in the KD+HBO_2_T mice likely enhanced the efficacy of this combined therapy.

A recent report by Listanti, et. al proposed that tumor-associated fibroblasts produce ketone bodies for cancer cells to use as fuel [68]. The authors have previously published several papers with similar findings [69], [70], [71]. In these studies, the authors created immortalized fibroblasts which were altered to overexpress rate-limiting enzymes in ketone body production, and co-cultured these cells with human breast cancer cells altered to overexpress enzymes involved in ketone body utilization. While this phenomenon may occur in the genetically altered culture system used by the authors, there is no evidence that this occurs naturally in cancer cells *in vitro* or in tumors *in vivo.* On the contrary, the literature as a whole strongly suggests that cancer cells cannot effectively use ketones for fuel. As described, most cancers do not express the SCOT enzyme which is necessary for ketone body utilization [65], [66]. Several studies have reported a deficiency of cancer cells to metabolize ketone bodies *in vitro* [24], [25]. Furthermore, it is widely accepted that ketone bodies are produced nearly exclusively from fatty acid β-oxidation in the liver. There is no known metabolic pathway by which fibroblasts can produce ketone bodies from glucose. Without additional compelling evidence to support these claims, we remain proponents of the notion that cancer cells cannot utilize ketone bodies as efficient energy substrates.

Potential concern may arise regarding the use of a diet therapy for cancer patients susceptible to cachexia. While low carbohydrate or ketogenic diets promote weight loss in overweight individuals, they are also known to spare muscle wasting during conditions of energy restriction and starvation [72], [73], [74], [75]. In an animal model of cancer cachexia, administration of a low carbohydrate, high fat diet prevented weight loss of the animals while simultaneously decreasing tumor size [76]. Similar effects were described in human cancer patients [64], [77]. The anti-cachexia effects of the KD are not surprising when considering a metabolic switch to fat metabolism and subsequent ketosis evolved as a method of sparing protein during prolonged fasting or starvation [72], [78], [79]. It makes sense that dietary-induced therapeutic ketosis in a cancer patient would prevent muscle wasting similarly as it does with athletes undergoing intense exercise [80]. Furthermore, when given as an adjuvant treatment to advanced cancer patients, the KD improves quality of life and enhances the efficacy of chemotherapy treatment in the clinic [81], [82]. This and other emerging evidence calls into question the common medical advice of limiting fat consumption in overweight cancer patients [83].

Veech and colleagues described the mechanisms by which ketone metabolism protects cells from oxidative damage [74], [78], while more recent evidence suggests that ketones function as HDAC inhibitors [84]. βHB metabolism results in an increased reduction of the NAD couple and increased oxidation of the co-enzyme Q inside the mitochondria. Increased oxidation of Q decreases semiquinone levels, subsequently decreasing superoxide anion production [74]. Increased reduction of the NADP couple enhances regeneration of reduced glutathione, an important endogenous antioxidant [74]. Thus, ketone body metabolism protects cells from oxidative damage by decreasing ROS production and by enhancing endogenous antioxidant capabilities. As previously discussed, cancer cells are unable to effectively metabolize ketone bodies; therefore, we do not expect that ketones would confer the same protective effects onto the cancer cell. HBO_2_T increases ROS production within the cell which can lead to membrane lipid peroxidation and cell death [85]. Cancer cells with mitochondrial damage and chaotic perfusion naturally produce chronically elevated levels of ROS but are susceptible to oxidative damage-induced cell death with even modest increases in ROS [58], [86]. We propose a potential mechanism of KD+HBO_2_T efficacy: the KD weakens cancer cells by glucose restriction and the inherent anti-cancer effects of ketone bodies while simultaneously conferring a protective advantage to the healthy tissue capable of ketone metabolism. This metabolic targeting sensitizes the cancer cells to HBO_2_T-induced ROS production and oxidative damage, contributing to the efficacy of combining KD with HBO_2_T. Additionally, ketone metabolism by the healthy tissues likely confers protection against the potential negative consequences of HBO_2_T (CNS oxygen toxicity) [87], [88], [89]. Recent *in vivo* studies support the neuroprotective effects of ketone esters [90], [91]. These hypothetical mechanisms may contribute to the safety and efficacy of the KD+HBO_2_T combined therapy.

Stuhr and Moen recently published a comprehensive review of the literature on the use of HBO_2_T for cancer [59]. The authors concluded that HBO_2_T should be considered a safe treatment for patients with varying malignancies and that there is no convincing evidence its use promotes cancer progression or recurrence. In the literature, there are a substantial number of studies indicating that HBO_2_T can induce marked anti-cancer effects *in vitro* and in animal and human studies alike [58], [59], [92]. Evidence is mixed, however, as other studies reported no effect with HBO_2_T [58], [59]. Indeed, in our present study, HBO_2_T alone did not improve the outcome of VM mice with metastatic cancer, but combining HBO_2_T with KD elicited a dramatic therapeutic effect. Perhaps adding the KD or another metabolic therapy (e.g. 2-deoxyglucose, 3-bromopyruvate) to HBO_2_T would produce similar results in these previously reported studies demonstrating no efficacy due to HBO_2_T alone. It is important to look for synergistic interactions between therapies which may increase the efficacy of cancer treatment. Scheck and coworkers reported complete remission without recurrence in 9 of 11 mice with glioma by combining the KD with radiation [93]. Marsh, et al. reported synergy between the restricted ketogenic diet and the glycolysis inhibitor 2-deoxyglucose [94]. Might adding HBO_2_T to these combination therapies elicit even better results? Similarly, might the use of adjuvant therapies like KD and HBO_2_T enhance patient response to standard care?

Our study strongly suggests that combining a KD with HBO_2_T may be an effective non-toxic therapy for the treatment of metastatic cancer. The efficacy of combining these non-toxic treatments should be further studied to determine their potential for clinical use. Based on the reported evidence, it is highly likely that these therapies would not only contribute to cancer treatment on their own, but might also enhance the efficacy of current standard of care and improve the outcome of patients with metastatic disease.
